# The fungus among us: Rice blast fungus blocks ROS production and starch breakdown to disrupt host resistance

**DOI:** 10.1093/plcell/koaf110

**Published:** 2025-05-05

**Authors:** Vicky Howe

**Affiliations:** Assistant Features Editor, The Plant Cell, American Society of Plant Biologists

Pathogens and their hosts are locked in an eternal battle: the pathogen attacks, the host defends. The pathogen evolves a more sophisticated attack strategy, the host develops a more advanced counter-attack. Over time, this leads to complex and multi-faceted host-pathogen interactions as each side vies for victory. In plants, chloroplasts are central to plant immunity, synthesizing various signaling and defense compounds in response to pathogenic invasion, including reactive oxygen species (ROS). The electron transport chains in both PSI and PSII provide the electrons required to form free radicals, such as superoxide anions (O_2_^•−^) (later converted to hydrogen peroxide [H_2_O_2_]), and singlet oxygen (^1^O_2_), which can cause both direct damage to pathogens and act as signaling molecules to elicit further immune responses (Kretschmer et al. 2019). To counter this, many pathogens secrete enzymes that detoxify ROS produced by the host.

In a recent study by Muxing Liu and coauthors (Liu et al. 2025), one such enzyme, the ascorbate peroxidase MoApx1, was identified in the rice blast fungus *Magnaporthe oryzae*. The authors showed that upon infection, secreted MoApx1 neutralizes H_2_O_2_ in rice (*Oryza sativa*). However, *M. oryzae* lacking functional MoApx1 was less effective in colonizing rice plants, suggesting MoApx1 enhanced the fungus's virulence. As previous studies indicated that successful infection requires more than just neutralizing ROS (Schouten et al. 2002; Robbertse et al. 2003), the group began to suspect that this peroxidase perhaps did more than just scavenge H_2_O_2_.

To find out what MoApx1 was doing, the researchers needed to find out where MoApx1 was going and what it was interacting with. Using GFP-tagged MoApx1, Liu and colleagues showed that MoApx1-GFP is first secreted into the cytoplasm of host cells by *M. oryzae* and then makes its way to the rice chloroplast. A yeast 2-hybrid screen against a rice cDNA library suggested MoApx1 might interact with several chloroplast proteins, including a PSI subunit, OsPsaD. This interaction was confirmed in vivo using co-immunoprecipitation in rice protoplasts. Using a split-YFP assay, the researchers showed that MoApx1 and OsPsaD indeed interact predominantly in the chloroplast.

The group next used AlphaFold3 to predict the interaction interface of these 2 proteins, which they confirmed using mutational analysis. Interestingly, this interaction site is also required for OsPsaD to bind to OsPsaC, another PSI subunit involved in the electron transport chain. Using an in vitro binding assay, the group showed that MoApx1 out-competes OsPsaC in binding to OsPsaD, inhibiting the electron transport chain and thereby reducing ROS production. But that's not all!

Looking again at the AlphaFold predictions of MoApx1 structure, the researchers could see that there were 2 β-sheet–rich domains at the C terminus that were not involved in binding to OsPsaD. When they searched the Protein Data Bank for similar structures, the authors found they resembled the starch-binding domain of the *Rhizopus oryzae* fungus. Given MoApx1's localization in the chloroplast, where starch is synthesized, it was plausible that these could be bona fide starch-binding domains. Indeed, MoApx1 could bind to starch in vitro! Moreover, in rice overexpressing the fungal MoApx1 protein, starch degradation was inhibited. As plants require starch to be broken down into glucose before utilizing it as an energy source, inhibiting starch hydrolysis deprives the plant of the energy it needs to fight the infection. It turns out that MoApx1 is a 3-in-1 enzyme: it (1) neutralizes H_2_O_2_; (2) binds to the PSI subunit OsPsaD to inhibit both photosynthesis and further ROS production; and (3) prevents starch hydrolysis to disrupt the host's energy supply, further weakening the plant's resistance to the fungus (Figure).

**Figure.**
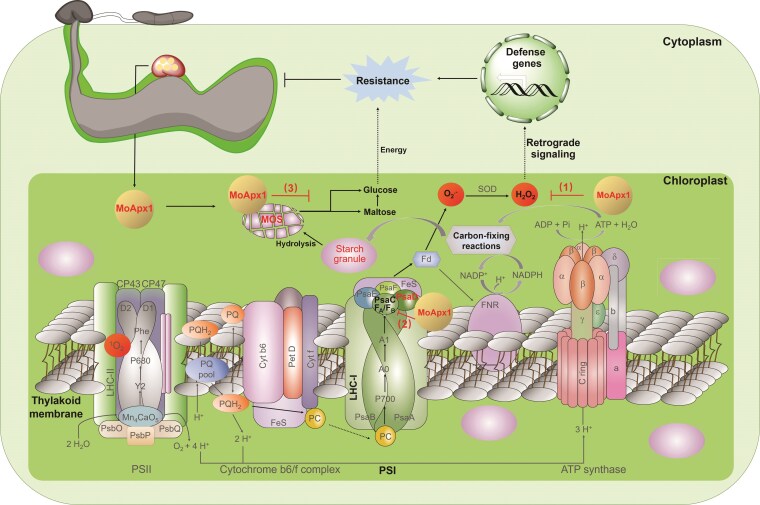
MoApx1 has multiple functions during host infection. (1) MoApx1 is secreted by *M. oryzae* and neutralizes host H_2_O_2_; (2) MoApx1 inhibits further ROS production by interfering with the PSI electron transport chain; and (3) MoApxI prevents starch breakdown by binding directly to starch, limiting the plant's energy supply and diminishing its ability to resist attack. Reprinted from Liu et al. (2025), Figure 8.

While many previous studies have focused on the ROS-neutralizing capabilities of secreted fungal effectors, this study is one of the few that suggests these enzymes may have more functions, acting in multiple pathways to disrupt host defenses. Who would have guessed a peroxidase would also bind to the photosystem complex AND starch? Clearly, this is an area of research that warrants further inspection. How many other pathogen effector proteins are similarly launching these multi-faceted attacks? And, more importantly, how can we develop crops that have equally sophisticated counter-attack strategies?

## Recent related articles in *The Plant Cell*

- Yan et al. 2023 used transcriptional profiling to identify hundreds of effector proteins employed by the rice blast fungus to enable successful infection.
- Ma et al. 2024 demonstrate how an ROS-activated kinase links pattern recognition–mediated ROS bursts with MAP kinase pathway activation and disease resistance.
- Oliveira-Garcia et al. 2023 show how the rice blast fungus coopts the host's clathrin-mediated endocytosis pathway to enable the uptake of fungal effectors into host cells.
